# Conserved Function of Fibrillin5 in the Plastoquinone-9 Biosynthetic Pathway in Arabidopsis and Rice

**DOI:** 10.3389/fpls.2017.01197

**Published:** 2017-07-13

**Authors:** Eun-Ha Kim, Dae-Woo Lee, Kyeong-Ryeol Lee, Su-Jin Jung, Jong-Seong Jeon, Hyun Uk Kim

**Affiliations:** ^1^Department of Agricultural Biotechnology, National Institute of Agricultural Science, Rural Development Administration Jeonju, South Korea; ^2^Graduate School of Biotechnology, Kyung Hee University Yongin, South Korea; ^3^Department of Bioindustry and Bioresource Engineering, Plant Engineering Research Institute, Sejong University Seoul, South Korea

**Keywords:** fibrillin, photosynthesis, plastoquinone, rice, solanesyl diphosphate synthase

## Abstract

Plastoquinone-9 (PQ-9) is essential for plant growth and development. Recently, we found that fibrillin5 (FBN5), a plastid lipid binding protein, is an essential structural component of the PQ-9 biosynthetic pathway in Arabidopsis. To investigate the functional conservation of FBN5 in monocots and eudicots, we identified *OsFBN5*, the Arabidopsis *FBN5* (*AtFBN5*) ortholog in rice (*Oryza sativa*). Homozygous *Osfbn5-1* and *Osfbn5-2* Tos17 insertion null mutants were smaller than wild type (WT) plants when grown on Murashige and Skoog (MS) medium and died quickly when transplanted to soil in a greenhouse. They accumulated significantly less PQ-9 than WT plants, whereas chlorophyll and carotenoid contents were only mildly affected. The reduced PQ-9 content of the mutants was consistent with their lower maximum photosynthetic efficiency, especially under high light. Overexpression of *OsFBN5* complemented the seedling lethal phenotype of the Arabidopsis *fbn5-1* mutant and restored PQ-9 and PC-8 (plastochromanol-8) to levels comparable to those in WT Arabidopsis plants. Protein interaction experiments in yeast and mesophyll cells confirmed that OsFBN5 interacts with the rice solanesyl diphosphate synthase OsSPS2 and also with Arabidopsis AtSPS1 and AtSPS2. Our data thus indicate that *OsFBN5* is the functional equivalent of *AtFBN5* and also suggest that the SPSs–FBN5 complex for synthesis of the solanesyl diphosphate tail in PQ-9 is well conserved in Arabidopsis and rice.

## Introduction

In plants, plastoquinone-9 (PQ-9) plays indispensable roles in plant growth and development. PQ-9 is an essential prenylquinone and functions as a mobile redox carrier in the photosynthetic electron transport chain (Trebst, 1978). Furthermore, the redox state of the PQ-9 pool regulates many physiological and molecular processes during short-term and long-term photoacclimations. These processes include phosphorylation of thylakoid membrane proteins (Vener et al., 1997; Zito et al., 1999; Depège et al., 2003); chloroplast expression of photosystem (PS) I and II genes (Allen, 1995; Maxwell et al., 1995; Pfannschmidt et al., 2001); and expression of nuclear-encoded genes such as ascorbate peroxidase (Karpinski et al., 1997), plastocyanin transcription factors (Adamiec et al., 2008), and others (Bräutigam et al., 2009; Akhtar et al., 2010). PQ-9 also participates in the biosynthesis of carotenoids as a cofactor for the desaturation of phytoene and ζ-carotene (Mayer et al., 1990; Norris et al., 1995). Reduced PQ shows antioxidant activity under abiotic and biotic stresses (Mubarakshina and Ivanov, 2010; Yadav et al., 2010; Kruk et al., 2016). Moreover, reduced PQ has been shown to act as a scavenger of toxic oxygen species generated in the thylakoid membranes and in *Chlamydomonas* under strong illumination stress (Hundal et al., 1995; Kruk and Trebst, 2008; Nowicka and Kruk, 2012). Furthermore, rapid oxidation of PQH_2_ to PQ and PQ degradation have been shown to occur under heavy metal and high light stresses (Kruk and Szymańska, 2012; Nowicka et al., 2016). *Solanesyl-diphosphate synthase 1* (*SPS1*)-overexpressing Arabidopsis lines with elevated levels of PQ-9 and its derivative plastochromanol-8 (PC-8) were shown to be more resistant to photo-oxidative stress than their wild type (WT) counterpart (Ksas et al., 2015). Moreover, when tobacco (*Nicotiana tabacum*) and black nightshade (*Solanum nigrum*) were challenged with the pathogens tobacco mosaic virus (TMV) and *Phytophthora infestans*, respectively, the contents of PQ-9 were increased (Maciejewska et al., 2002; Bajda et al., 2009).

Genes involved in the PQ-9 pathway in plants have been intensively studied using combined genomic, genetic, and biochemical approaches (Cheng et al., 2003; Sadre et al., 2006; Block et al., 2013; Liu and Lu, 2016). The PQ-9 biosynthetic pathway consists of two stages: first, the benzene quinone ring and prenyl side chain precursors are synthesized, followed by condensation of the benzene quinone ring and prenyl side chain. Next, subsequent modifications occur. The benzene quinone ring PQ-9 precursor is homogentisic acid (HGA; Hutson and Threlfall, 1980). This compound is synthesized from tyrosine by the catalytic action of tyrosine aminotransferase (TAT) and 4-hydroxyphenylpyruvate reductase (HPPR). The prenyl side chain of PQ-9 is derived from glyceraldehyde 3-phosphate (G3P) and pyruvate through the 2-C-methyl-D-erythritol 4-phosphate (MEP) pathway (Disch et al., 1998). The solanesyl moiety is generated by *trans*-type consecutive condensation of isopentenyl diphosphate (IPP; C5) and its isomer dimethylallyl diphosphate (DMAPP; C5), which are synthesized through the MEP and MVA pathways with geranylgeranyl diphosphate (GGPP; C20). This process is catalyzed by solanesyl diphosphate synthases (SPSs) (Hirooka et al., 2003, 2005).

Database mining of fully sequenced genomes has shown that duplication of plastidic isoform SPSs is widespread in land plants such as Arabidopsis, rice (*Oryza sativa*), maize (*Zea mays*), and soybean (*Glycine max*) (Block et al., 2013). The SPS genes for the synthesis of the PQ-9 solanesyl diphosphate (SPP; C45) moiety have been identified and functionally characterized in Arabidopsis (*AtSPS1* and *AtSPS2*), tomato (*SlSPS*), rice (*OsSPS2:LOC_Os05g50550*), and *Hevea brasiliensis* (*HbSDS*) (Jun et al., 2004; Phatthiya et al., 2007; Ohara et al., 2010; Block et al., 2013; Jones et al., 2013). The two Arabidopsis SPS enzymes AtSPS1 and SPS2 are targeted to chloroplasts and are responsible for the biosynthesis of PQ-9 (Block et al., 2013). These two enzymes have been shown to function as homodimers (Hirooka et al., 2003; Jun et al., 2004; Hsieh et al., 2011). In rice, OsSPS2 is localized in plastids and is involved in the production of PQ-9 SPP (Ohara et al., 2010). OsSPS3 has been shown to be present in rice and to share amino acid sequence similarity with OsSPS2 (Block et al., 2013). Although the function of OsSPS3 has not yet been reported, it might also be involved in PQ-9 formation (Liu and Lu, 2016). Condensation of HGA and SPP is the first committed step in PQ-9 biosynthesis. This step is catalyzed by homogentisate solanesyltransferase (HST) and produces the intermediate 2-methyl-6-solanesyl-1,4-benzoquinol (MSBQ), which is then methylated by a methyltransferase (VTE3) to form PQ-9 in plants. PQ-9 is cyclized into PC-8 by tocopherol cyclase (VTE1) (Savidge et al., 2002; Sattler et al., 2003). In chloroplasts, tocopherol synthesis and PQ-9 synthesis are closely related. HGA is the common head group of PQ-9 and tocopherols. In tocopherol biosynthesis, condensation of HGA with phytyl diphosphate by homogentisate phytyltransferase (VTE2) yields 2-methyl-6-phytyl-1,4-benzoquinol (Collakova and DellaPenna, 2001).

Fibrillins (FBNs), which are lipid-associated proteins, are found in all organisms performing oxygenic photosynthesis (Pozueta-Romerso et al., 1997; Kessler et al., 1999; Simkin et al., 2007; Cunningham et al., 2010; Heinnickel and Grossman, 2013; Lohscheider and Bártulos, 2016). In higher plants, FBN can be distinguished into 11 subfamilies (Singh and McNellis, 2011). New FBNs were recently identified in algae by searching FBN sequences from publicly available algal genomes (Lohscheider and Bártulos, 2016). Proteomic studies of plastid subcompartments have identified 12 known FBNs in Arabidopsis. Seven FBNs are strongly enriched in plastoglobules (PGs), while other FBNs are distributed throughout the stroma or are associated with the thylakoid membranes (Lundquist et al., 2012). Singh and McNellis (2011) showed that FBNs contain a conserved lipocalin “motif 1” in the N-terminal region and conserved residues in the C-terminal region, including aspartic acid. FBNs have been predicted to adopt a three-dimensional β-barrel structure with a small α-helical lid, similar to that of lipocalin (Lohscheider and Bártulos, 2016). This structure indicates that FBNs are involved in the binding and transport of small hydrophobic molecules (Flower, 1996; Singh and McNellis, 2011).

FBNs are mainly found in chromoplasts, PGs, and algal eyespots and have important roles in the formation of fibril structures in these organelles (Duruère et al., 1994; Rey et al., 2000; Singh et al., 2010). Group 1 members have been reported to be involved in PG formation and thylakoid maintenance (Rey et al., 2000; Simkin et al., 2007). Group 4 members have also been shown to be involved in PG formation. Furthermore, FBNs are involved in growth and development, tolerance to oxidative stress, and hormone signaling (Leitner-Dagan et al., 2006; Singh et al., 2010). The cyanobacterium *Synechocystis pgl1/pgl2* mutant exhibits altered thylakoid ultrastructure, reduced pigment levels, and is more susceptible to light (Cunningham et al., 2010). Group 1 and 2 members are involved in hormone signaling such as abscisic acid-mediated, jasmonate-mediated, and gibberellin-mediated responses to abiotic stress (Yang et al., 2006; Youssef et al., 2009). FBN4 has been suggested to be involved in the transport of PQ-9 between thylakoids and PG, an idea supported by the finding that FBN4 deficiency in apple and Arabidopsis plants resulted in reduced tolerance to abiotic and biotic stresses (Singh et al., 2010, 2012). In addition, we recently showed that FBN5 is involved in PQ-9 biosynthesis in Arabidopsis. Arabidopsis mutant plants containing low levels of *FBN5-B* accumulated less PQ-9 and PC-8, leading to their increased susceptibility to cold stress and lower photosynthetic performance. FBN5-B interacted with SPS1 and SPS2 in chloroplasts. It was hypothesized that FBN5-B stimulates the enzymatic activity of SPS1 and SPS2 by binding to the hydrophobic solanesyl moiety and helping to release this moiety from the enzymes in Arabidopsis (Kim et al., 2015).

Although FBNs are presumed to play significant roles in photosynthetic organisms, they have been identified and characterized in only a few plant species to date (e.g., Arabidopsis, cucumber, tomato, and pepper) (Singh and McNellis, 2011). Rice is a major cereal. Thus, characterization of FBNs function in rice would increase our knowledge about maintaining photosynthetic efficiency and stress tolerance. In this study, we identified an Arabidopsis FBN5 (AtFBN5) ortholog, OsFBN5, in rice and investigated its function in two rice *Osfbn5* Tos17 insertion mutants. We found that deficiency of OsFBN5 resulted in reduced levels of PQ-9 and PC-8 in the leaves and increased susceptibility to excess light energy. Moreover, OsFBN5 interacted with AtSPS1, AtSPS2, and OsSPS2. OsFBN5 was also capable of complementing AtFBN5 function when expressed in the *fbn5-1* Arabidopsis mutant. The contents of PQ-9 and PC-8 present in complemented mutants were almost indistinguishable from those in WT Arabidopsis plants. These results provide strong evidence that the function of FBN5 in PQ-9 biosynthesis is well conserved between eudicots and monocots.

## Materials and Methods

### Plant Materials and Growth Conditions

The *Arabidopsis thaliana* Columbia-0 ecotype (WT) and the *AtFBN5*/*fbn5* T-DNA insertion mutant (Salk_064597), the latter of which harbors a disruption in *FBN5* (At5g09820), were grown in soil or agar plates containing 0.5× MS (Murashige and Skoog) medium supplemented with/without sucrose. Plants were propagated under a 16 h light/8 h dark photoperiod with 100 μmol m^-2^ s^-1^ fluorescence light at 22°C. Two rice Tos17 insertion mutant alleles, *Osfbn5-1* (ND8652) and *Osfbn5-2* (NG2517) (cultivar Nipponbare), were isolated from the Rice Tos17 Insertion Mutant Database^1^. Rice seeds were germinated on 0.5× MS agar medium supplemented with 3% sucrose. The plants were grown at 28°C for 7 days under 100 μmol m^-2^ s^-1^ light intensity. Uniformly grown seedlings were transferred into fresh water or transplanted onto soil in the growth chamber and grown with 100 or 600 μmol m^-2^ s^-1^ light at 28°C.

### PCR Analysis

The genotypes of the *Osfbn5* mutant alleles, the Arabidopsis *fbn5-1* (*Atfbn5-1*) mutant, and the complemented lines were determined by genomic DNA PCR analysis with a genotyping primer set (Supplementary Table 1). Total RNA was extracted from the leaf tissues using an RNeasy kit (Qiagen), which included a DNase treatment step. cDNA was synthesized from total RNA using RNA to cDNA EcoDry^TM^ premix (Clontech, CA, United States) following the manufacturer’s instructions. The PCR reactions were performed with Takara Ex Taq DNA polymerase and an RT-PCR primer set (Supplementary Table 1).

### Analysis of Prenyl-Lipids

To analyze the PQ-9, PC-8, tocopherol, carotenoid, and chlorophyll contents of the plants, total lipids were extracted and analyzed with HPLC using a Shimadzu LC-20AD chromatography unit as previously described (Kim et al., 2015). Chromatography was conducted at 30°C on a C18 reverse-phase column (5 μM Supelco Discovery C18 column, 250 × 4.6 mm). Total lipids were extracted from frozen tissue grindates in liquid nitrogen in cold ethyl acetate. After centrifugation, the supernatant was transferred to a new tube and evaporated under nitrogen. The extract was redissolved in 95% ethanol. PQ-9, PC-8, and tocopherols were analyzed with an isocratic solvent system consisting of methanol/hexane (9:1, v/v) at a flow rate of 1.0 mL min^-1^. PQ-9 was detected by absorption at 255 nm, while PC-8 and tocopherols were detected fluorimetrically (290 nm excitation and 330 nm emission). The compounds were quantified by comparison to their corresponding external calibration standards, and the data were corrected by comparison with the recovery of rac-Tocol (Matreya, PA, United States) as the internal standard.

For the analysis of carotenoids and chlorophylls, the chromatographic conditions were the same as above. Solvent A (acetonitrile:water = 9:1 v/v with 0.1% triethylamine) and solvent B (ethyl acetate) were used with the following gradient: 0 to 5 min, 0 to 33.3% B; 5 to 33 min, 33.3 to 66.7% B; 33 to 33.5 min, 66.7 to 100% B; 33.5 to 38 min, 100% B; 38 to 38.5 min, 0% B; and 38.5 to 43 min, 0% B. The HPLC peak areas at 440 nm were integrated.

### Yeast Two-Hybrid Assay

The coding sequences for *OsFBN5* and *OsSPS2* without the predicted chloroplast transit peptide regions were amplified from rice leaf cDNA with the yeast two-hybrid primer set (Supplementary Table 1). The amplified products were cloned into the entry vector pENTR^TM^/D-TOPO^®^ (Invitrogen, CA, United States) and then subcloned into two destination vectors, pDEST-GBKT7 (bait) and pDEST-GADT7 (prey) using the Gateway system. The resulting bait and prey vectors, together with the AtFBN5, AtSPS1, and AtSPS2 vectors (Kim et al., 2015), were introduced into the yeast strains PBN204 (containing *URA3*, *ADE2*, and *lacZ* as reporters) and AH109 (containing *HIS3*, *ADE2*, and *lacZ*). Transformants were spotted onto synthetic defined medium lacking Leu and Trp (SD-LW) or also lacking Ura (SD-LWU), Ade (SD-LWA), or Ade and His (SD-LWAH). After incubation for 3 days at 30°C, the colonies were replica-plated onto several selective media. As a negative control, cells transformed with parental bait (pGBKT7) and prey (pGADT7) vectors (Clontech, CA, United States) were used. Cells transformed with the SV40 large T-antigen (pGBKT7) and murine p53 (pGADT7) vectors were used as a positive control.

### BiFC Assay

The coding sequences for *OsFBN5* and *OsSPS2* without stop codons were amplified with the bimolecular fluorescence complementation (BiFC) primer set (Supplementary Table 1). The amplified products were cloned into pENTR^TM^/D-TOPO^®^ (Invitrogen, CA, United States) and then inserted into the pJJ2536 (containing the N-terminus of YFP) and pJJ2537 (containing the C-terminus of YFP) destination vectors for the BiFC assay. To assay transient expression, maize mesophyll protoplasts (5 × 10^5^ cells per sample) were isolated from the second leaves of etiolated plants. The vectors were delivered into maize mesophyll protoplasts using the polyethylene glycol-calcium mediated method, followed by 12–16 h incubation to allow transient expression (Cho et al., 2009). Chlorophyll autofluorescence was used as a chloroplast marker. Expression of the fusion constructs was monitored using a confocal microscope (LSM 510 META, Carl Zeiss).

### Complementation of *Atfbn5-1* with *OsFBN5*

To produce the complementation constructs, the full coding sequence of *OsFBN5* was amplified with the complementation primer pairs (Supplementary Table 1). The amplicon was cloned into the entry vector pENTR^TM^/D-TOPO^®^ and then subcloned into the destination vector pB2GW7 (Karimi et al., 2002) using the Gateway system. The resulting construct was transformed into heterozygous *FBN5/fbn5-1* Arabidopsis plants by *Agrobacterium* (GV3101) mediation using the floral dip transformation method (Clough and Bent, 1998). Transformants were selected via their resistance to BASTA (Bayer). Genomic DNA was extracted from the leaves of the BASTA-resistant plants and used to identify the homozygous *fbn5-1* plants harboring *35S:OsFBN5* via PCR. *OsFBN5* expression was detected in the complemented *fbn5-1* plants using the appropriate primer pairs (Supplementary Table 1). *Actin2* was used as an internal control.

### Measurement of Photosynthetic Parameters

A portable chlorophyll fluorimeter (Walz) was used to measure the maximal photochemical activity of PSII (*F*_v_/*F*_m_) under atmospheric conditions. Prior to *F*_v_/*F*_m_ measurement, leaves were adapted to the dark for 10 min. *F*_v_/*F*_m_ was calculated as (*F*_m_ -*F*_o_)/*F*_m_, where *F*_o_ is the initial chlorophyll fluorescence level, and *F*_m_ is the maximal fluorescence level, determined with an intense pulse of white light. The stress-dependent reduction in *F*_v_/*F*_m_ was interpreted as the photoinhibition of PSII.

### Phylogenetic Analysis

FBN orthologs in rice were identified by similarity searches using BLAST^2^ and phytozome v10.1^3^. The protein sequences of the individual FBN family members from Arabidopsis were used as queries against the rice genome. Sequence alignment was performed using the default ClustalW parameters and maximum likelihood was used for tree construction in MEGA6 program.

### Accession Numbers

Sequence data from this article can be found in the GenBank databases under the following accession numbers: Rice FBN5 (OsFBN5), AK070660; rice SPS1 (OsSPS1), AK09456; rice SPS2 (OsSPS2), AK066579; rice SPS3 (OsSPS3), XM_015764714.

## Results

### Identification of FBN5 Homologs from Rice

**FIGURE 1:**
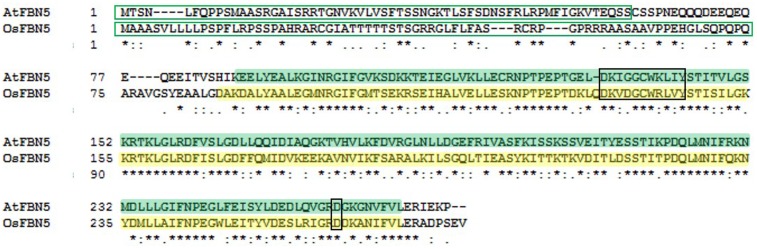
Sequence alignment of AtFBN5 (At5g09820, 273 aa) and OsFBN5 (LOC_Os04g34460, 278 aa). Plastid targeting peptides (green box) were predicted by the ChloroP program. AtFBN5 and OsFBN5 share 60% identical residues. The well conserved lipocalin “motif 1” and well conserved aspartic acid residue are marked in black boxes. The AtFBN5 and OsFBN5 plastid lipid-associated domains are shaded in green and yellow, respectively. Asterisks indicate identical residues between Arabidopsis and rice.

Previously, we proposed a novel function for FBN5 in the PQ-9 biosynthetic pathway of Arabidopsis (Kim et al., 2015). To investigate the roles of FBNs in rice, a monocot model species, a phylogenetic tree was constructed based on amino acid sequences of FBNs from Arabidopsis and rice. Arabidopsis and rice express 14 and 11 FBN genes, respectively (Supplementary Figure 1). Analysis of putative physicochemical properties, isoelectric points (PIs), and hydrophobicities of the Arabidopsis and rice FBN sequences after removal of the predicted plastid targeting peptides revealed predicted similar physicochemical properties between the Arabidopsis and rice FBN homologs (Supplementary Figure 2). Each FBN Arabidopsis/rice homolog pair showed more similar PIs than hydrophobicities. While the *FBN1*, *FBN3*, and *FBN7* genes are duplicated in Arabidopsis, each of these genes is unique in rice. AtFBN5 and OsFBN5 shared 60% amino acid identity after removal of the predicted plastid targeting peptides (**Figure 1**). In Arabidopsis, an alternatively spliced transcript of *FBN5*, AtFBN5-A, could not interact with SPS (Kim et al., 2015). The product of this transcript was altered in 14 amino acids, including a 10 amino acid deletion at the C-terminus, indicating that these residues are indispensable for AtFBN5 function. These residues are well conserved in OsFBN5 (**Figure 1**), suggesting that OsFBN5 can bind SPS. A fairly well conserved lipocalin motif 1 was present in the Arabidopsis (DKIGGCWKLIY) and rice (DKVDGCWRLVY) FBN5s. Aspartic acid residues in the C-terminal regions, which are highly conserved in the FBN family, are present in Arabidopsis and rice FBNs (Singh and McNellis, 2011). The high amino acid sequence similarity of OsFBN5 and AtFBN5 suggests that OsFBN5 might function as a structural protein providing a scaffold for prenyl chain assembly during the synthesis of PQ-9 in rice, similar to Arabidopsis AtFBN5.

### Isolation and Characterization of FBN5 Knockout Rice Mutants

**FIGURE 2:**
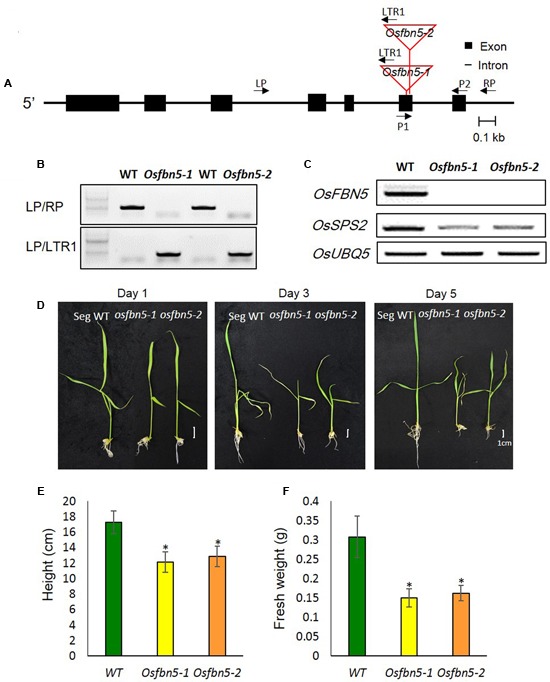
Isolation and growth phenotypes of the *OsFBN5* mutant alleles. **(A)** Schematic diagram of the rice *FBN5* gene with the Tos17 insertion. Black boxes and black lines represent exons and introns, respectively. The Tos17 insertion sites for two independent mutant lines, *Osfbn5-1* and *Osfbn5-2*, are indicated by red lines. Arrows with names indicate primers for PCR experiments. **(B)** Genomic DNA PCR analysis of *Osfbn5-1* and *Osfbn5-2* progeny plants. A 1.1-kb PCR product was detected using the LP+RP primers, but not the LTR1+RP primers, in WT segregants. In knockout mutants, 830 bp and 850 bp products were detected using the LTR1+LP primers, but not the LP+RP primers. A representative sample from each indicated genotype is shown. **(C)** RT-PCR analysis of *OsFBN5* and *OsSPS2* expression in segregant wild type (WT), *Osfbn5-1*, and *Osfbn5-2* mutant lines. The *OsFBN5* transcript was amplified by primers P1+P2. A product was detected in WT segregants, but not in *Osfbn5-1* or *Osfbn5-2* plants. *OsUBQ5* was used as a PCR loading control. **(D)** Photographs of WT, *Osfbn5-1*, and *Osfbn5-2* seedling plants at 1, 3, and 5 days after transferring 7-day-old plants into water. **(E)** Plant height and **(F)** fresh weight were measured on the 5th day in water. Each data point represents the mean (± SD) of five different plants. The asterisk indicates a significant difference between WT and mutant plants (^∗^*P* < 0.05; Student’s *t*-test).

We identified two Tos17 insertion mutant alleles in the sixth exon of OsFBN5 (*LOC_Os04g34460*), *Osfbn5-1* and *Osfbn5-2*, and confirmed their insertions by genomic DNA sequencing (**Figure 2A**). Progeny seeds from the self-fertilized heterozygous plants, designated *OsFBN5/Osfbn5-1* and *OsFBN5/Osfbn5-2*, were germinated on agar medium supplemented with 3% sucrose. Genotype analysis of growing seedlings by genomic DNA PCR with *OsFBN5*-specific and Tos17-specific primers revealed nearly normal segregation of Mendelian inheritance (**Figure 2B** and Supplementary Table 2). RT-PCR of RNA isolated from the selected homozygous plant showed that no *OsFBN5* transcripts had accumulated (**Figure 2C**). Segregant WT and homozygote plants were grown on MS medium supplemented with 3% sucrose for one week and then transferred to water for growth observation. Unlike the *Atfbn5-1* plants, the *Osfbn5* homozygous mutant plants were not seedling lethal. However, they were smaller than the WT plants (**Figures 2D–F**). When the WT and *Osfbn5* homozygous mutant plants were transplanted onto soil in the greenhouse, the mutant plants rapidly dried and eventually died, while the WT plants grew and yielded seeds.

**FIGURE 3:**
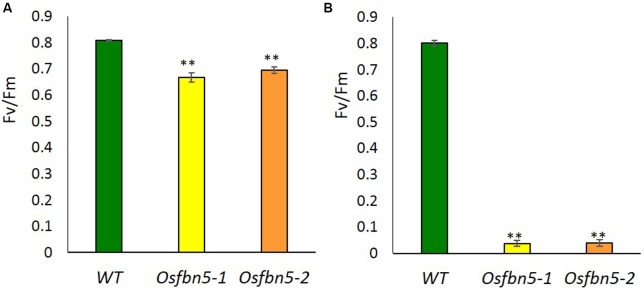
Photosystem II photoinhibition (*F*_v_/*F*_m_) of segregant WT and *Osfbn5* mutant plants. Rice plants were grown for 7 days under 100 μmol m^-2^ s^-1^ light intensity on 0.5× MS medium supplemented with 3% sucrose. Next, the plants were transferred to soil in a growth chamber and grown under a light intensity of **(A)** 100 μmol m^-2^ s^-1^ or **(B)** 600 μmol m^-2^ s^-1^. Chlorophyll fluorescence was measured in the plant leaves at 5 days after the transfer. Each data point represents the mean (±SD) of five different plants. The asterisks indicate a significant difference between WT and *Osfbn5* mutant plants (^∗∗^*P* < 0.01; Student’s *t*-test).

The maximum efficiency of PSII photochemistry was measured by the chlorophyll fluorescence parameter *F*_v_/*F*_m_. To this end, plants were transferred to soil under 100 or 600 μmol m^-2^ s^-1^ light after 7 days growth on MS medium under 100 μmol m^-2^ s^-1^ light. Photoinactivation of *Osfbn5* homozygous mutant plants was light intensity dependent (**Figure 3**). The *Osfbn5* homozygous mutant plants were dramatically photoinhibited, with an *F*_v_/*F*_m_ value of 0.05 under 600 μmol m^-2^ s^-1^ light compared to 100 μmol m^-2^ s^-1^ light, while WT plants were not compromised under either condition and showed 0.8 units of photochemical activity.

### *OsFBN5* Mutation Reduces the Levels of PQ-9 and PC-8

**FIGURE 4:**
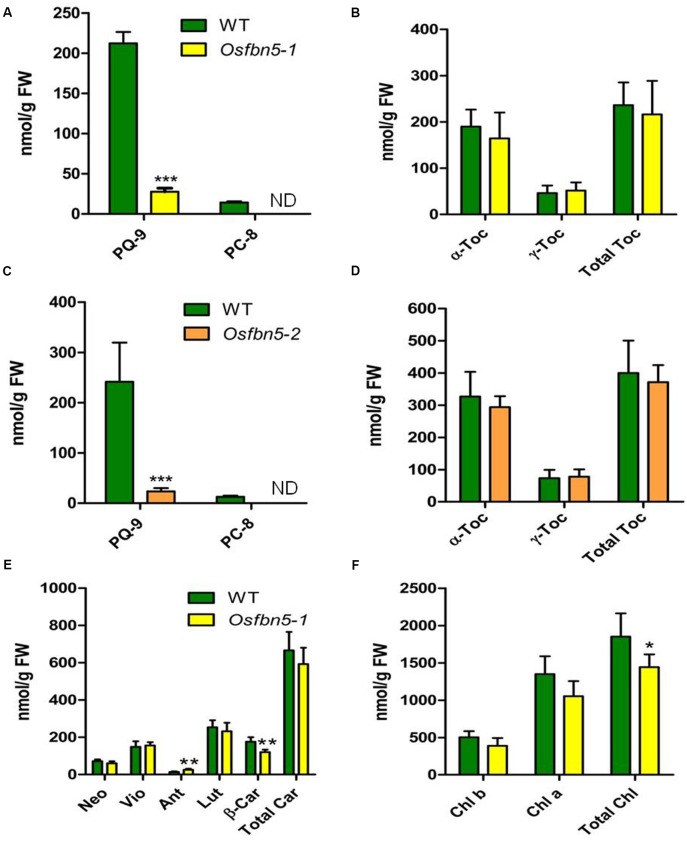
Quantification of PQ-9 and PC-8 **(A,C)**, and tocochromanols **(B,D)** in segregant WT, *Osfbn5-1*, and *Osfbn5-2* mutant plants and quantification of carotenoid **(E)** and chlorophylls **(F)** in segregant WT and *Osfbn5-1* mutant plants. Reversed-phase HPLC was used to analyze total lipids from the leaves 1 day after the 7-day-old plants were transferred into water. The quantities of individual components were determined relative to standards. Asterisks represent significance relative to WT. ^∗^*P* < 0.05, ^∗∗^*P* < 0.01, and ^∗∗∗^*P* < 0.001 (Student’s *t*-test). Data are means ± SD (*n* = 3–5). ND, not detected. Toc, tocopherol; Neo, neoxanthin; Vio, violaxanthin; Ant, antheraxanthin; Lut, lutein; β-Car, β-carotene; Car, carotene; Chl, chlorophyll.

Since AtFBN5 is involved in the PQ-9 biosynthetic pathway (Kim et al., 2015), we next used HPLC to determine the amounts of PQ-9, PC-8, tocochromanols, carotenoids, and chlorophyll in the mutant and WT plants. The amount of PQ-9 was reduced about sevenfold in the leaves of *Osfbn5-1* and *Osfbn5-2* mutant plants compared with the amount in WT plants (**Figures 4A,C**). PC-8, the product of PC-9 cyclization, was not detectable in either of the mutants, in contrast to WT plants (**Figures 4A,C**). In the *Osfbn5-1* and *Osfbn5-2* mutant plants, the levels of γ-tocopherol, α-tocopherol, and total tocopherols were not significantly different (**Figures 4B,D**). The level of β-carotene in the *Osfbn5-1* plants was reduced by about 30% compared with that in the WT plants, whereas the levels of other carotenoids were not significantly reduced in *Osfbn5-1* than WT plants (**Figure 4E**). The level of antheraxanthin was increased in the *Osfbn5-1* plants (**Figure 4E**), indicating that the mutant plants were more photostressed than the WT plants. Both chlorophyll *a* and *b* levels were slightly reduced in the *Osfbn5-1* plants compared with the WT plants (**Figure 4F**).

### OsFBN5 Interacts with SPSs from Rice and Arabidopsis

**FIGURE 5:**
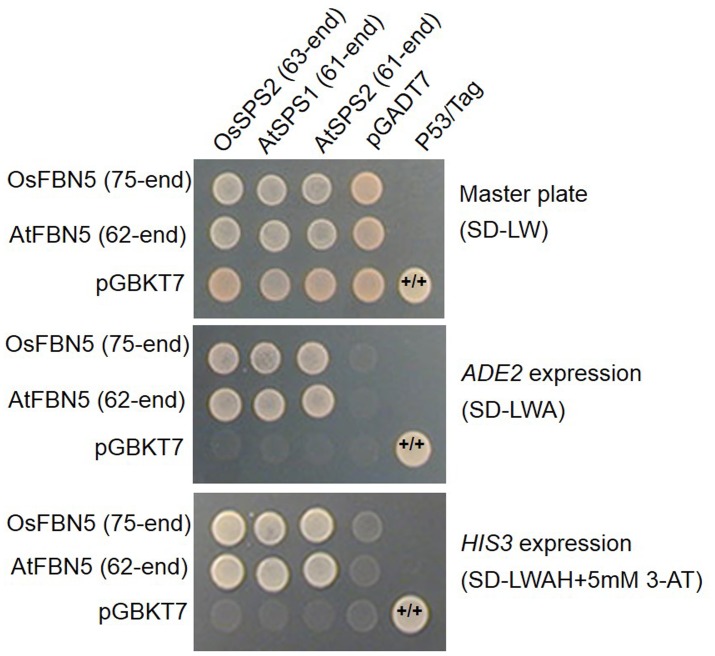
Yeast two-hybrid assay to test the strength of the interaction of OsFBN5 and AtFBN5 with SPS enzymes from rice and Arabidopsis. AH109 transformants were dropped onto selective medium lacking Leu and Trp (SD-LW) or also lacking Ade (SD-LWA) or Ade and His (SD-LWAH), with or without 5 mM 3-amino-1,2,3-triazole (3-AT), a competitive inhibitor of yeast HIS3. Positive control, yeast transformed with the P53 bait plasmid and the Tag prey plasmid. Negative control, yeast transformed with the parental bait vector (pGBKT7) and the prey vector (pGADT7).

**FIGURE 6:**
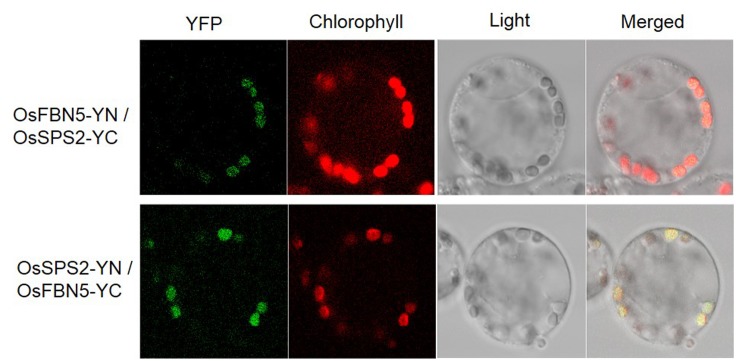
Visualization of the OsFBN5-OsSPS2 interaction in maize protoplasts by the BiFC assay. Maize mesophyll protoplasts were cotransfected with recombinant vectors containing the indicated constructs. Chlorophyll autofluorescence was used as a chloroplast marker. Fluorescence was observed 12–16 h after transfection.

OsSPS2 has been shown to provide an SPP for PQ-9 formation in rice (Ohara et al., 2010). Therefore, we tested whether OsFBN5 interacts with OsSPS2 using the yeast two-hybrid system. After confirming that the fusion product of the mature OsFBN5 cDNA and the GAL4 DNA binding domain (BD fusion) did not retain transcriptional activation ability, this fusion protein was used as the bait. We also tested interactions of OsFBN5 and OsSPS2 with their Arabidopsis counterpart. OsFBN5 interacted with AtSPS1 and AtSPS2, as well as with OsSPS2 in both AH109 cells and PBN204 cells (**Figure 5** and Supplementary Figure 3). In addition, AtFBN5 interacted with OsSPS2 in both yeast lines (**Figure 5** and Supplementary Figure 3). We next tested the strength of the interaction between the proteins in AH109 cells on medium containing 3-amino-1,2,3-triazole, a competitive inhibitor of yeast HIS3. These experiments revealed that the interactions of OsFBN5 and AtFBN5 with OsSPS2, AtSPS1, and AtSPS2 were as strong as those of the positive control proteins (**Figure 5**). Yeast growth and β-galactosidase activity assays in PBN204 cells showed less strong interaction of OsFBN5 with OsSPS2 and AtSPS1 than with AtSPS2 (Supplementary Figure 3). OsSPS2 exhibits 77.5 and 76.6% identity with AtSPS1 and AtSPS2, respectively, after removal of the chloroplast targeting peptide (Supplementary Figure 4). Interestingly, the transcript levels of *OsSPS2* were reduced in the *Osfbn5* homozygous mutant plants than WT (**Figure 2C**). The OsFBN5–OsSPS2 interaction was further confirmed by BiFC experiments in maize mesophyll protoplasts. The YFP signals overlapped well with the chlorophyll autofluorescence signal (**Figure 6**), suggesting that these two proteins interact with each other in chloroplasts.

### Complementation of the *Atfbn5-1* Mutant with the Rice Ortholog of FBN5

**FIGURE 7:**
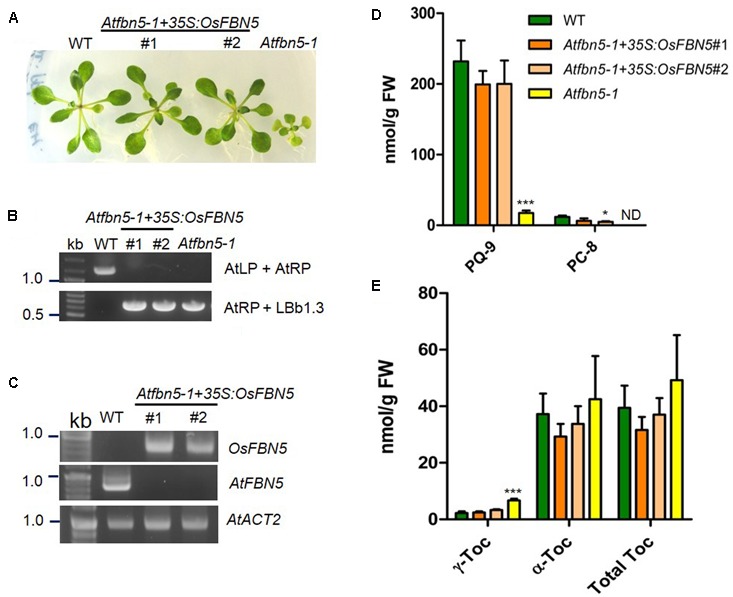
Complementation of Arabidopsis *fbn5-1* mutants by *OsFBN5. OsFBN5* was expressed under control of the CaMV 35S promoter. Plants were grown on MS medium supplemented with 2% sucrose. **(A)** Phenotypes of WT plants, the #1 and #2 complemented lines, and *Atfbn5-1* plants. Expression of *OsFBN5* rescues the seedling lethal phenotype of the *Atfbn5-1* mutant. **(B)** Genomic DNA PCR genotyping of WT and the Arabidopsis *fbn5-1* insertion mutant using primers AtLP, AtRP, and LBb1.3. **(C)** RT-PCR analysis of the expression of *OsFBN5* and *AtFBN5* in WT plants and in the #1 and #2 complemented lines. *OsFBN5* transcripts amplified by the F+R primers (Supplementary Table 1) were detected in the #1 and #2 complemented lines but not in WT plants. The *AtFBN5* transcript amplified by the F+R primers (Supplementary Table 1) was only detected in WT plants. *AtACT2* was used as a PCR loading control. **(D)** PQ-9 and PC-8 contents and **(E)** tocopherol contents in leaves from WT plants, #1 and #2 complemented lines, and *Atfbn5-1* plants. Contents were analyzed by reverse-phase HPLC. Asterisks represent significance relative to WT. ^∗^*P* < 0.05 and ^∗∗∗^*P* < 0.001 (student’s *t*-test). Data are mean ± SD (*n* = 3–5). ND, not detected.

To test the relevance of the OsFBN5 and AtSPSs interactions and to determine whether rice FBN5 is capable of functionally replacing AtFBN5, *OsFBN5* cDNA was constitutively expressed under the CaMV 35S promoter in the *fbn5-1* Arabidopsis mutant. Two *Atfbn5-1* + *35S:OsFBN5* transgenic complemented lines (#1 and #2), each of which contained *OsFBN5* cDNA and was homozygous for *Atfbn5-1*, were identified by genomic DNA PCR (**Figures 7A,B**). RT-PCR analysis showed that *OsFBN5* was expressed in the *Atfbn5-1* transgenic plants but not in their WT counterpart (**Figure 7C**). The *Atfbn5-1* + *35S:OsFBN5* lines displayed a WT phenotype (**Figure 7A**), indicating that rice OsFBN5 in *fbn5-1* Arabidopsis plants has a function similar to that of AtFBN5 in WT Arabidopsis plants. The levels of PQ-9 and PC-8 in the *Atfbn5-1* + *35S:OsFBN5* lines, which were deficient in *Atfbn5-1*, were comparable to the levels in WT plants (**Figure 7D**). Moreover, the levels of total tocopherols in the complemented Arabidopsis plants were similar to the levels in WT plants (**Figure 7E**). The levels of γ-tocopherol in the complemented Arabidopsis plants, which were elevated in *Atfbn5-1* plants, were recovered to those of WT plants. These results demonstrate that rice OsFBN5 is able to complement the *Atfbn5-1* mutant plants, suggesting the similar function of OsFBN5 with AtFBN5 in Arabidopsis PQ-9 biosynthesis.

## Discussion

We have previously shown that a plastid lipid-associated protein, FBN5, is required for PQ-9 biosynthesis in Arabidopsis through physical and functional interactions with SPS (Kim et al., 2015). Our working hypothesis is that FBN5 binds to hydrophobic solanesyl moieties generated by SPS1 and SPS2 in FBN5/SPS homodimeric complexes and removes these moieties from the enzyme active sites to catalyze the reaction and maintain substrate turnover. In the present study, we proposed that FBN5 function in PQ-9 synthesis is not limited to Arabidopsis and is well conserved in rice, a monocot.

FBNs are present in all oxygenic photosynthetic organisms and were recently described as “green cut proteins,” referring to a group of proteins proposed to be necessary for optimal photosynthetic processes (Heinnickel and Grossman, 2013). Although FBNs appear to be involved in important processes in higher plants, their functions in rice are not known. Here, we show that the FBN family is well conserved in rice (Supplementary Figure 1), and that the physiochemical properties of FBN family members are very similar to those of their Arabidopsis orthologs, especially PI (Supplementary Figure 2). The localization of each rice FBN protein is expected to be consistent with the PG, stromal, and thylakoid membrane localizations of the orthologous Arabidopsis FBN proteins identified in a previous proteomics study. Moreover, FBN localization in Arabidopsis is correlated with PI (Lundquist et al., 2012).

OsFBN5 physically interacts with OsSPS2 (**Figures 5**, **6**), suggesting involvement of FBN5 in SPP moiety synthesis. Yeast growth and β-galactosidase activity assays in PBN204 cells suggested less strong interaction of OsFBN5 with OsSPS2 and AtSPS1 than with AtSPS2 (Supplementary Figure 3). However, this is likely attributed from other reasons such as lower expression of OsSPS2 and AtSPS1 in PBN204 cells rather than less strong interactions of them with OsFBN5. Because the yeast growth in AH109 cells indicated that the interactions of OsFBN5 with OsSPS2 and AtSPS1 were as strong as that of OsFBN5 and AtSPS2. In addition, the strength test of the interactions between the proteins in AH109 cells against a competitive inhibitor of yeast HIS3 showed similar strength between OsFBN5 with OsSPS2, AtSPS1 and AtSPS2 (**Figure 5**). The high degree of sequence conservation (over 60% identity) between Arabidopsis and rice FBN5 and SPS suggests that OsFBN5 is also able to physically interact with Arabidopsis SPS1 and SPS2, like AtFBN5 (**Figure 5** and Supplementary Figure 4). The ability of rice FBN5 to complement the *fbn5-1* mutation in Arabidopsis indicates that OsFBN5 interacts with AtSPS1 and AtSPS2, thus participating in PQ-9 synthesis in Arabidopsis chloroplasts (**Figure 7**).

Similar to Arabidopsis (Block et al., 2013), rice also expresses three SPS genes, *OsSPS1* (*LOC_Os06g46450*), *OsSPS2*, and *OsSPS3* (*LOC_Os12g17320*) (Supplementary Figures 4, 5). OsSPS1 and OsSPS2 have been shown to be responsible for ubiquinone-9 and PQ-9 SPP synthesis, respectively (Ohara et al., 2010; Ducluzeau et al., 2011). OsSPS3 is predicted to be targeted to chloroplasts, shares 90% identity with OsSPS2 (Supplementary Figure 4), and is phylogenetically closer to OsSPS2 than OsSPS1 (Block et al., 2013). Examination of *OsSPS1*, *OsSPS2*, and *OsSPS3* expression data in different organs available in the Rice Genome Annotation Project public expression database^4^ showed that *OsSPS2* and *OsSPS3* are highly expressed in leaves, while *OsSPS1* is mainly expressed in embryos, pistils, and anthers (Supplementary Figure 5). Although OsSPS3 activity for SPP synthesis has not yet been reported, OsSPS3 is presumed to be a paralog of OsSPS2, raising the possibility that it can also contribute to PQ-9 biosynthesis. *OsSPS2* might be a major contributor to PQ-9 biosynthetic flux since its transcript level was higher than that of *OsSPS3* in leaf tissue (Supplementary Figure 5). Moreover, the high amino acid sequence similarity of OsSPS3 with AtSPS1 and AtSPS2 (Supplementary Figure 4) also suggests that OsSPS3 may interact with AtFBN5 and OsFBN5.

FBN functions appear to be conserved among plant species. For instance, Arabidopsis FBN4 mutants and FBN4 knockdown apple trees showed increased sensitivity to various oxidative stresses and alteration in the accumulation of osmophilic material inside PG, implying that FBN4 is involved in plant stress tolerance and trafficking of hydrophobic molecules between thylakoids and PGs (Singh et al., 2010). In addition, FBN group 1 genes are involved in disease resistance. Knockdown of *LeChrC* (*FBN1*) gene expression in tomato (Leitner-Dagan et al., 2006) and knockdown of *FBN1b* gene expression in Arabidopsis (Cooper et al., 2003) resulted in increased susceptibility to the fungus *Botrytis cinerea* and the bacterium *Pseudomonas syringae* pv. *maculicola*, respectively. Furthermore, silencing of the *C40.4* (*FBN1*) gene in potato resulted in stunted growth, decreased tuber yield, and reduced non-photochemical quenching (Monte et al., 1999) and overexpression of bell pepper *FBN1* resulted in increased plant height under stress conditions (Rey et al., 2000). These results suggest that FBN group 1 genes are related to modulation of photosynthetic efficiency and plant development upon stress.

We found that the *Osfbn5* mutants displayed about sevenfold lower levels of PQ-9 compared to the WT plants and an undetectable amount of PC-8 (**Figures 4A,C**). *Osfbn5* mutants exhibited slow growth and lower photosynthetic efficiency under normal growth conditions (**Figures 2**, **3**), demonstrating that PQ-9 is required for plant growth and optimal photosynthetic performance. When *Osfbn5* mutant plants were exposed to high light conditions, the PQ-9 defect was exacerbated, leading to photodamage of PSII and death (**Figure 3**). The PQ-9 deficiency in the *Osfbn5* mutants is likely the cause of the reduced chlorophyll and carotenoid contents, but not the tocopherol contents (**Figure 4**). These features of *Osfbn5* are consistent with previous studies performed with PQ-9-deficient plants such as *Atfbn5-1*, *atsps1*, *atsps2*, and *atsps1atsps2* (Block et al., 2013; Kim et al., 2015). By monitoring the decay of radioactivity incorporated into PQ-9 from a labeled precursor, the half-life of PQ-9 was shown to be very short in spinach cells (Wanke et al., 2000). Furthermore, upon sudden exposure of Arabidopsis plants to excessively high light conditions, total PQ-9 contents were dramatically decreased. This effect was followed by a progressive increase, with almost fourfold enhanced accumulation compared to PQ levels measured before stress at the end of the time course (Ksas et al., 2015). Recent studies focused on PQ-9 antioxidant functions, thus expanding the PQ-9 role as a photosynthetic electron carrier between PSII and PSI and a redox gene regulator (Nowicka and Kruk, 2012; Ksas et al., 2015; Kruk et al., 2016). Therefore, the capacity of PQ-9 synthesis must be optimized in order to maintain stable yet dynamic PQ-9 concentrations to enable suitable physiological responses (Szymańska and Kruk, 2010), as supported by our OsFBN5 results.

Recently, Lohscheider and Bártulos (2016) reported the distribution and numbers of FBNs in some algae and higher plants that perform oxygenic photosynthesis. Interestingly, some algae belonging to Chlorophyta such as *Ostreococcus tauri* and *Micromonas pusilla* do not contain FBN5 orthologs, while *Chlamydomonas reinhardtii* does. We also identified a few plant species that do not contain FBN5 orthologs (data not shown). It would be interesting to determine whether these organisms have alternative structural proteins that function like FBN5 or whether their SPS enzymes contain a structural domain for binding to the SPP moiety for PQ-9 synthesis.

## Author Contributions

E-HK, D-WL, K-RL, and S-JJ performed the experiments; E-HK, D-WL, K-RL, J-SJ, and HUK analyzed the data; and E-HK, D-WL, J-SJ, and HUK wrote the paper. All authors read and approved the final manuscript.

## Conflict of Interest Statement

The authors declare that the research was conducted in the absence of any commercial or financial relationships that could be construed as a potential conflict of interest.

## Footnotes

1 https://tos.nias.affrc.go.jp/

2 www.ncbi.nlm.nih.gov/BLAST/

3 phytozome.jgi.doe.gov

4 http://rice.plantbiology.msu.edu

## References

[B1] Adamiec M. Drath M. Jackowski G. (2008). Redox state of plastoquinone pool regulates expression of *Arabidopsis thaliana* genes in response to elevated irradiance. *Acta Biochim. Pol.* 55 161–173. 18231654

[B2] Akhtar T. A. Lees H. A. Lampi M. A. Enstone D. Branin R. A. Greenberg B. M. (2010). Photosynthetic redox imbalance influences flavonoid biosynthesis in *Lemna gibba*. *Plant Cell Environ.* 33 1205–1219. 10.1111/j.1365-3040.2010.02140.x 20199616

[B3] Allen J. F. (1995). Thylakoid protein phosphorylation, state 1–state 2 transitions and photosystem stoichiometry adjustment: redox control at multiple level of gene expression. *Physiol. Plant.* 93 196–205. 10.1034/j.1399-3054.1995.930128.x

[B4] Bajda A. Konopka-Postupolska D. Krzymowska M. Hennig J. Skorupinska-Tudek K. Surmacz L. (2009). Role of polyisoprenoids in tobacco resistance against biotic stresses. *Physiol. Plant.* 135 351–364. 10.111/j.1399-3054.2009.01204.x 19292825

[B5] Block A. Fristedt R. Rogers S. Kumar J. Barnes B. Elowsky C. G. (2013). Functional modeling identifies paralogous solanesyl-diphosphate synthases that assemble the side chain of plastoquinone-9 in plastids. *J. Biol. Chem.* 288 27594–27606. 10.1074/jbc.M113.492769 23913686 PMC3779756

[B6] Bräutigam K. Dietzel L. Kleine T. Ströher E. Wormuth D. Dietz K.-J. (2009). Dynamic plastid redox signals integrate gene expression and metabolism to induce distinct metabolic states in photosynthetic acclimation in *Arabidopsis*. *Plant Cell* 21 2715–2732. 10.1105/tpc.108.062018 19737978 PMC2768923

[B7] Cheng Z. Sattler S. Maeda H. Sakuragi Y. Bryant D. A. DellaPenna D. (2003). Highly divergent methyltransferases catalyze a conserved reaction in tocopherol and plastoquinone synthesis in cyanobacteria and photosynthetic eukaryotes. *Plant Cell* 15 2343–2356. 10.1015/tpc.013656 14508009 PMC197300

[B8] Cho J. I. Ryoo N. Eom J. S. Lee D. W. Kim H. B. Jeong S. W. (2009). Role of the rice hexokinases *OsHXK5* and *OsHXK6* as glucose sensors. *Plant Physiol.* 149 745–759. 10.1104/pp.108.131227 19010999 PMC2633841

[B9] Clough S. Bent A. (1998). Floral dip: a simplified method for *Agrobacterium*-mediated transformation of *Arabidopsis thaliana*. *Plant J.* 16 735–743. 10.1046/j.1365-313x.1998.00343.x 10069079

[B10] Collakova E. DellaPenna D. (2001). Isolation and functional analysis of homogentisate phytyltransferase from *Synechocystis* sp. PCC6803 and Arabidopsis. *Plant Phsysiol.* 127 1113–1124. 10.1104/pp.010421 PMC129280 11706191

[B11] Cooper B. Clarke J. D. Budworth P. Kreps J. Hutchison D. Park S. (2003). A network of rice genes associated with stress response and seed development. *Proc. Natl. Acad. Sci. U.S.A.* 100 4945–4959. 10.1073/pnas.0737574100 12684538 PMC153660

[B12] Cunningham F. X. Jr. Tice A. B. Pham C. Gantt E. (2010). Inactiation of genes encoding plastoglobuli-like proteins in *Synechocystis* sp. PCC 6803 leads to a light-sensitive phenotype. *J. Bacteriol.* 192 1700–1709. 10.1128/JB.01434-09 20081034 PMC2832526

[B13] Depège N. Bellafiore S. Rochaix J.-D. (2003). Role of chloroplast protein kinase Stt7 in LHCII phosphorylation and state transition in *Chlamydomonas*. *Science* 299 1572–1575. 10.1126/science.1081397 12624266

[B14] Disch A. Hemmerlin A. Bach T. J. Rohmer M. (1998). Mevalonate-derived isopentenyl diphosphate is the biosynthetic precursor of ubiquinone prenyl side chain in tobacco BY-2 cells. *Biochem. J.* 331(Pt 1), 615–621. 10.1042/bj3310615 PMC1219396 9531505

[B15] Ducluzeau A.-L. Wamboldt Y. Elowsky C. G. Mackenzie S. A. Schuurink R. C. Basset G. J. C. (2011). Gene network reconstruction identifies the authentic *trans*-prenyl diphosphate synthase that makes the solanesyl moiety of ubiquinone-9 in Arabidopsis. *Plant J.* 69 366–375. 10.1111/j.1365-313X.2011.04796.x 21950843

[B16] Duruère J. Römer S. d’Harlingue A. Backhaus R. A. Kuntz M. Camara B. (1994). Fibril assembly and carotenoid overaccumulation in chromoplasts: a model for supramolecular lipoprotein structures. *Plant Cell* 6 119–133. 10.1105/tpc.6.1.119 8130642 PMC160421

[B17] Flower D. R. (1996). The lipocalin protein family: structure and function. *Biochem. J.* 318 (Pt 1), 1–14. 10.1042/bj3180001 PMC1217580 8761444

[B18] Heinnickel M. L. Grossman A. R. (2013). The GreenCut: re-evaluation of physiological role of previously studied proteins and potential novel protein functions. *Photosynth. Res.* 116 427–436. 10.1007/s11120-013-9882-6 23873414

[B19] Hirooka K. Bamba T. Fukusaki E. Kobayashi A. (2003). Cloning and kinetic characterization of *Arabidopsis thaliana* solanesyl diphosphate synthase. *Biochem. J.* 370 679–686. 10.1042/BJ20021311 12437513 PMC1223189

[B20] Hirooka K. Izumi Y. An C.-I. Nakazawa Y. Fukusaki E. Kobayshi A. (2005). Functional analysis of two solanesyl diphosphate synthases from *Arabidopsis thaliana*. *Biosci. Biotechnol. Biochem.* 69 592–601. 10.1271/bbb.69.592 15784989

[B21] Hsieh F. L. Chang P.-H. Ko T.-P. Wang A. H.-J. (2011). Structure and mechanism of an Arabidopsis medium/long-chain-length prenyl pyrophosphate synthase. *Plant Physiol.* 155 1079–1090. 10.1104/pp.110.168799 21220764 PMC3046570

[B22] Hundal T. Forsmark-Andreé P. Ernster L. Adersson B. (1995). Antioxidant activity of reduced plastoquinone in chloroplast thylakoid membranes. *Arch. Biochem. Biophys.* 324 117–122. 10.1006/abbi.1995.9920 7503545

[B23] Hutson K. G. Threlfall D. R. (1980). Synthesis of plastoquinone-9 and phytylplastoquinone from homogentisate in lettuce chloroplasts. *Biochim. Biophys. Acta* 632 630–648. 10.1016/0304-4165(80)90339-6 7002223

[B24] Jones M. O. Perez-Fons L. Robertson F. P. Bramley P. M. Fraser P. D. (2013). Functional characterization of long-chain prenyl diphosphate synthases from tomato. *Biochem. J.* 449 729–740. 10.1042/BJ20120988 23126257

[B25] Jun L. Saiki R. Tatsumi K. Nakagawa T. Kawamukia M. (2004). Identification and subcellular localization of two solanesyl diphosphate synthases from *Arabidopsis thaliana*. *Plant Cell Physiol.* 45 1882–1888. 10.1093/pcp/pch211 15653808

[B26] Karimi M. Inzé D. Depicker A. (2002). GATEWAY vectors for *Agrobacterium*-mediated plant transformation. *Trends Plant Sci.* 7 193–195. 10.1016/S1360-1385(02)02251-3 11992820

[B27] Karpinski S. Escobar C. Karpinska B. Creissen G. Mullineaux P. M. (1997). Photosynthetic electron transport regulates the expression of cytosolic ascorbate peroxidase genes in Arabidopsis during excess light stress. *Plant Cell* 9 627–640. 10.1105/tpc.9.4.627 9144965 PMC156944

[B28] Kessler F. Schnell D. Blobel G. (1999). Identification of proteins associated with plastoglobules isolated from pea (*Pisum sativum* L.) chloroplasts. *Planta* 208 107–113. 10.1007/s004250050540 10213003

[B29] Kim E.-H. Lee Y. Kim H. U. (2015). Fibrillin5 is essential for plastoquinone-9 biosynthesis by binding to solanesyl diphosphate synthases in Arabidopsis. *Plant Cell* 27 2956–2971. 10.1105/ptc.15.00707 26432861 PMC4682332

[B30] Kruk J. Szymańska R. (2012). Singlet oxygen and non-photochemical quenching contributes to oxidation of the plastoquinone-pool under light stress in *Arabidopsis*. *Biochim. Biophys. Acta* 1817 705–710. 10.1016/j.bbabio.2012.02.011 22365927

[B31] Kruk J. Szymańska R. Nowicka B. Dlużewska J. (2016). Function of isoprenoid quinones and chromanols during oxidative stress in plants. *New Biotechnol.* 33 636–643. 10.1016/j.nbt.2016.02.010 26970272

[B32] Kruk J. Trebst A. (2008). Plastoquinol as a singlet oxygen scavenge in photosystem II. *Biochim. Biophys. Acta* 1777 154–162. 10.1016/j.bbabio.2007.10.008 18005659

[B33] Ksas B. Becuwe N. Chevalier A. Havaux M. (2015). Plant tolerance to excess light energy and photooxidative damage relies on plastoquinone biosynthesis. *Sci. Rep.* 5:10919 10.1038/srep10919 PMC4454199 26039552

[B34] Leitner-Dagan Y. Ovadis M. Shklarman E. Elad Y. Ray David D. Vainstein A. (2006). Expression and functional analyses of the plastid lipid-associated protein CHRC suggest its role in chromoplastogenesis and stress. *Plant Physiol.* 142 233–234. 10.1104/pp.106.082404 16815957 PMC1557619

[B35] Liu M. Lu S. (2016). Plastoquinone and ubiquinone in plants: biosynthesis, physiological function and metabolic engineering. *Front. Plant Sci.* 7:1898 10.3389/fpls.2016.01898 PMC5159609 28018418

[B36] Lohscheider J. N. Bártulos C. R. (2016). Plastoglobules in algae: a comprehensive comparative study of the presence of major structural and functional components in complex plastids. *Mar. Genomics* 28 127–136. 10.1016/j.margen.2016/06.005 27373732

[B37] Lundquist P. K. Poliakov A. Bhuiyan N. H. Zybailov B. Sun Q. van Wijk K. J. (2012). The functional network of the Arabidopsis plastoglobule proteome based on quantitative proteomics and genome-wide coexpression analysis. *Plant Physiol.* 158 1172–1192. 10.1104/pp.111.193144 22274653 PMC3291262

[B38] Maciejewska U. Polkowska-Kowalczyk L. Swiezewska E. Szkopinska A. (2002). Plastoquinone: possible involvement in plant disease resistance. *Acta Biochim. Pol.* 49 775–780. 12422246

[B39] Maxwell D. P. Laudenbach D. E. Huner N. P. A. (1995). Redox regulation of light harvesting complex II and cab mRNA abundance in *Dunaliella salina*. *Plant Physiol.* 109 787–795. 10.1104/pp.109.3.787 12228633 PMC161378

[B40] Mayer M. P. Beyer P. Kleinig H. (1990). Quinone compounds are able to replace molecular oxygen as terminal electron acceptor in phytoene desaturation in chromoplasts of *Narcissus pseudonarcissus* L. *Eur. J. Biochem.* 191 359–363. 10.1111/j.1432-1033.1990.tb19130.x 2384084

[B41] Monte E. Ludevid D. Prat S. (1999). Leaf C40.4: a carotenoid-associated protein involved in the modulation of photosynthetic efficiency? *Plant J.* 19 339–410. 10.1046/j.1365-313X.1999.00537.x 10504562

[B42] Mubarakshina M. M. Ivanov B. N. (2010). The protection and scavenging of reactive oxygen species in the plastoquinone pool of chloroplast thylakoid membranes. *Physiol. Plant.* 140 103–110. 10.1111/j.1399-3054.2010.01391.x 20553418

[B43] Norris S. R. Barrette T. DellaPenna D. (1995). Genetic dissection of carotenoid synthesis in arabidopsis defines plastoquinone as an essential component of phytoene desaturation. *Plant Cell* 7 2139–2149. 10.1105/tpc.7.12.2139 8718624 PMC161068

[B44] Nowicka B. Kruk J. (2012). Plastoquinol is more active than a-tocopherol in singlet oxygen scavenging during high light stress of *Chlamydomonas reinhardtii*. *Biochim. Biophys. Acta* 1817 389–394. 10.1016/j.bbabio.20112.12.002 22192719

[B45] Nowicka B. Pluciński B. Kuczyńska P. Kruk J. (2016). Prenyllipid antioxidants participate in response to acute stress induced by heavy metals in green microalga *Chlamydomonas reinhardtii*. *Environ. Exp. Bot.* 123 98–107. 10.1016/j.envexpbot.2015.11.008

[B46] Ohara K. Sasaki K. Yazaki K. (2010). Two solanesyl diphosphate synthases with different subcellular localizations and their respective physiological roles in *Oryza sativa*. *J. Exp. Bot.* 61 2686–2692. 10.1093/jxb/erq103 PMC2882263 20421194

[B47] Pfannschmidt P. Schutze K. Brost M. Oelmuller R. (2001). A novel mechanism of nuclear photosynthesis gene regulation by redox signals from the chloroplast during photosystem stoichiometry adjustment. *J. Biol. Chem.* 276 36125–36130. 10.1074/jbc.M105701200 11468291

[B48] Phatthiya A. Takahashi S. Chareonthiphakorn N. Koyama T. Wititsuwannakul D. Wititsuwannakul R. (2007). Cloning and expression of the gene encoding solanesyl diphosphate synthase from *Hevea brasiliensis*. *Plant Sci.* 172 824–831. 10.1016/j.plantsce.2006.12.015

[B49] Pozueta-Romerso J. Pozueta-Romero J. Rafia F. Houlné G. Cheniclet C. Carde J. P. (1997). A ubiquitous plant housekeeping gene PAP, encodes a major protein component of bell pepper chromoplasts. *Plant Physiol.* 115 1185–1194. 10.1104/pp.115.3.1185 9390444 PMC158583

[B50] Rey P. Gillet B. Römer S. Eymery E. Massimino J. Peltier G. (2000). Over-expression of a pepper plastid lipid-associated protein in tobacco leads to changes in plastid ultrastructure and plant development upon stress. *Plant J.* 21 483–494. 10.1046/j.1365-313x.2000.00699.x 10758499

[B51] Sadre R. Gruber J. Frentzen M. (2006). Characterization of homogentisate prenyltransferases involved in plastoquinone-9 and tocochromanol biosynthesis. *FEBS Lett.* 580 5357–5362. 10.1046/j.1365-313x.2000.00699.x 16989822

[B52] Sattler S. E. Cahoon E. B. Coughlan S. J. DellaPenna D. (2003). Characterization of tocopherol cyclases from higher plants and cyanobacteria. Evolutionary implications for tocopherol synthesis and function. *Plant Cell* 132 42184–42195. 10.1104/pp.103.024257 PMC181302 12913173

[B53] Savidge B. Weiss J. D. Wong Y. H. Lassner M. W. Shewmaker C. K. Post-Beittenmiller D. (2002). Isolation and characterization of homogentisate phytyltransferase genes from *Synechocystis* sp. PCC6803 and Arabidopsis. *Plant Physiol.* 129 321–332. 10.1104/pp.010747 12011362 PMC155895

[B54] Simkin A. J. Gaffe J. Alcaraz J. P. Carde J. P. Bramley P. M. Fraser P. D. (2007). Fibrillin influence on plastid ultrastructure and pigment content in tomato fruit. *Phytochemistry* 68 1545–1556. 10.1016/j.phytochem.2007.03.014 17466343

[B55] Singh D. K. Laremore T. N. Smith P. B. Maximova S. N. Mcnellis T. W. (2012). Knockdown of *FIBRILLIN4* gene expression in apple decreases plastoglobule plastoquinone content. *PLoS ONE* 7:e47547 10.1371/journal.pone.004754 PMC3470590 23077632

[B56] Singh D. K. Maximova S. N. Jensen P. J. Lehman B. L. Ngugi H. K. McNellis T. W. (2010). *FIBRILLIN4* is required for plastoglobule development and stress resistance in Apple and Arabidopsis. *Plant Physiol.* 154 1281–1293. 10.1104/pp.110.164095 20813909 PMC2971606

[B57] Singh D. K. McNellis T. W. (2011). Fibrillin protein function: the tip of the iceberg? *Trends Plant Sci.* 16 432–441. 10.1016/j.tplants.2011.03.014 21571574

[B58] Szymańska R. Kruk J. (2010). Plastoquinol is the main prenyllipid synthesized during acclimation to high light conditions in Arabidopsis and is converted to plastochromanol by tocopherol cyclase. *Plant Cell Physiol.* 51 537–545. 10.1093/pcp/pcq017 20164151

[B59] Trebst A. (1978). Plastoquinones in photosynthesis. *Philos. Trans. R. Soc. Lond. B* 284 591–599. 10.1098/rstb.1978.0092

[B60] Vener A. V. Van Kan P. J. Rich P. R. Ohad I. Andersson B. (1997). Plastoquinol at the quinol oxidation site of reduced cytochrome bf mediates signal transduction between light and protein phosphorylation: thylakoid protein kinase deactivation by a single turnover flash. *Proc. Natl. Acad. Sci. U.S.A.* 94 1585–1590. 10.1073/pnas.94.4.1585 11038603 PMC19835

[B61] Wanke M. Swiezewska E. Dallner G. (2000). Half-life of ubiquinone and plastoquinone in spinach cells. *Plant Sci* 154 183–187. 10.1016/S0168-9452(00)00200-4 10729617

[B62] Yadav D. K. Kruk J. Sinha R. K. Pospišil P. (2010). Singlet oxygen scavenging activity of plastoquinol in photosystem II of higher plants: electron paramagnetic resonance spin-trapping study. *Biochim. Biophys. Acta* 1797 1587–1605. 10.1016/j.bbabio.2010.07.003 20637718

[B63] Yang Y. Sulpice R. Himmelbach A. Meinhard M. Christmann A. Grill E. (2006). Fibrillin expression is regulated by abscisic acid response regulators and is involved in abscisic acid-mediated photoprotection. *Proc. Natl. Acad. Sci. U.S.A.* 103 6061–6066. 10.1073/pnas.0501720103 16571665 PMC1458696

[B64] Youssef A. Laizet Y. Block M. A. Maréchal E. Alcaraz J.-P. Larson T. R. (2009). Plant lipid-associated fibrillin proteins condition jasmonate production under photosynthetic stress. *Plant J.* 61 436–445. 10.1111/j.1365-313X.2009.04067.x 19906042

[B65] Zito F. Finazzi G. Delosme R. Nitschke W. Picot D. Wollman F. A. (1999). The Qo site of cytochrome b6f complexes controls the activation of the LHCII kinases. *EMBO J.* 18 2961–2969. 10.1093/emboj/18.11.2961 10357809 PMC1171378

