# Supplementary material for: Conserved Function of Fibrillin5 in the Plastoquinone-9 Biosynthetic Pathway in Arabidopsis and Rice
Source: Front Plant Sci. 2017 Jul 13;8:1197. doi: 10.3389/fpls.2017.01197 (PMC5507956; doi:10.3389/fpls.2017.01197)
Supplement: Supplementary file 1 [file Presentation_1.PDF]

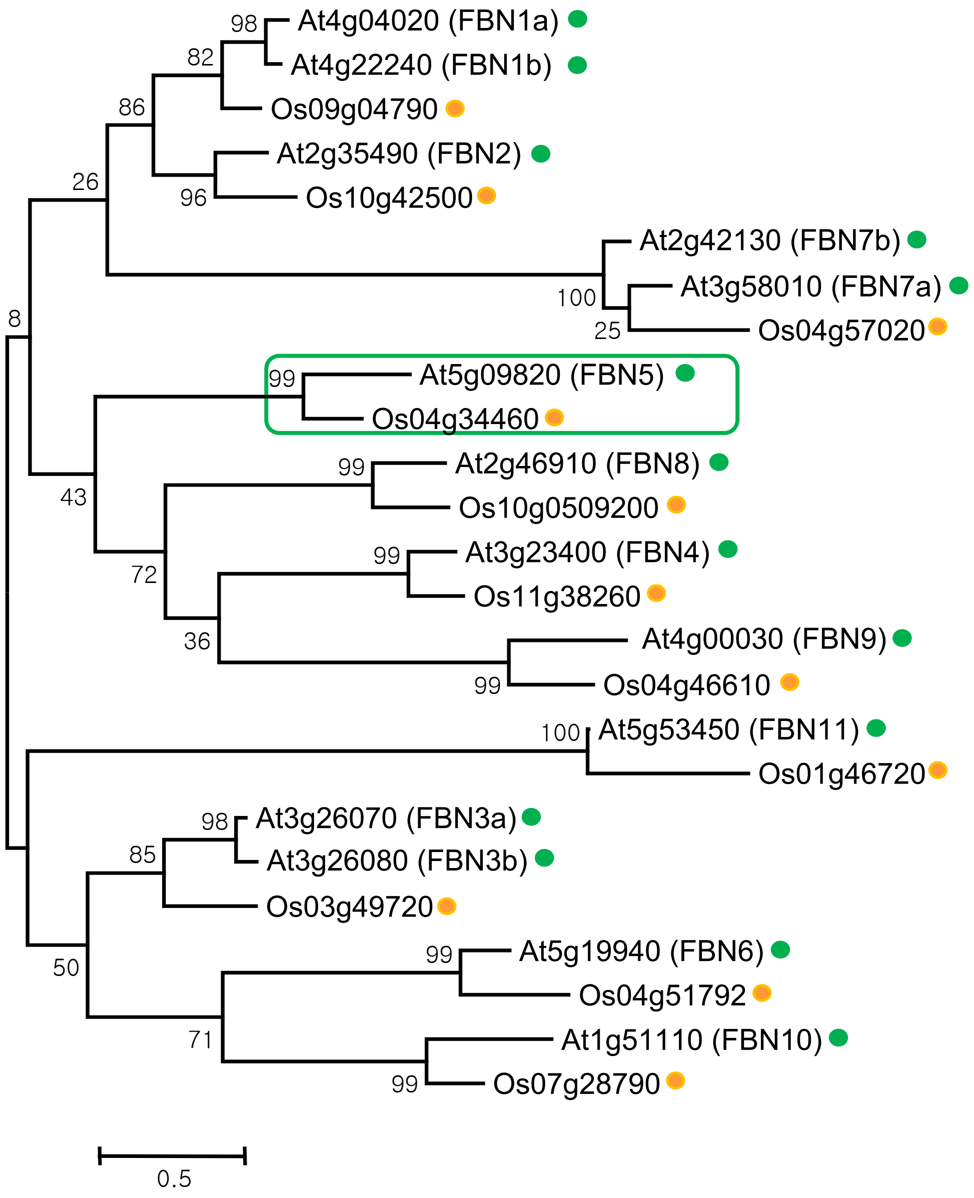

Supplementary Figure 1. Phylogenetic relationship of FBN proteins from Arabidopsis and rice. Sequence alignment was performed using the default ClustalW parameters and Maximum Likelihood was used for tree construction in MEGA6 software. Bootstrap values are given at the nodes as percentage of 1000 replicates. The scale bar indicates the number of amino acid substitutions per site.

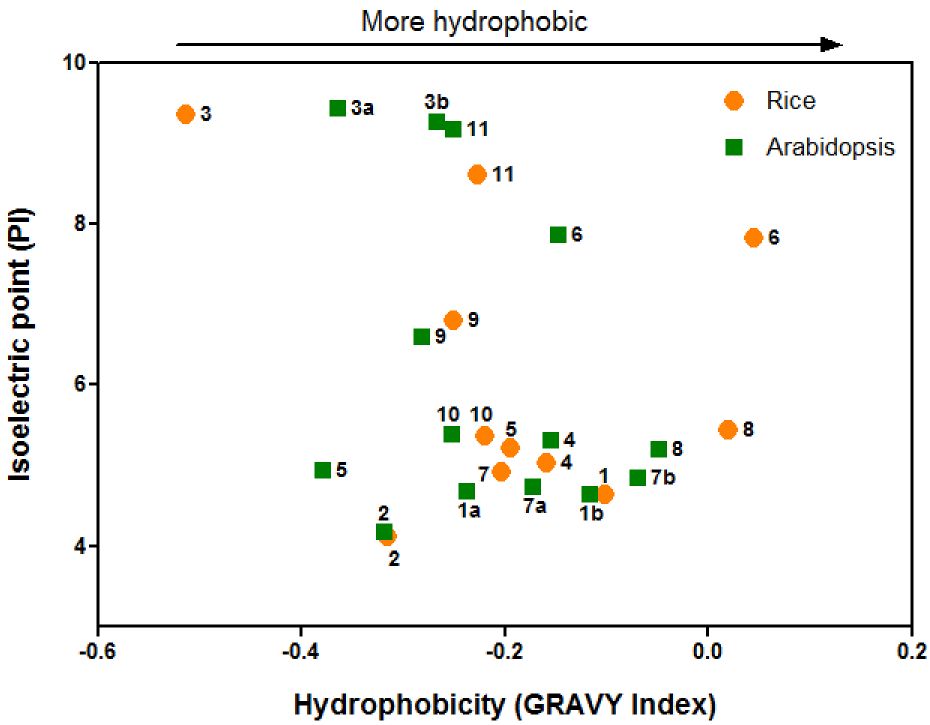

Supplementary Figure 2. Physicochemical properties of FBN proteins from Arabidopsis and rice. PI and hydrophobicity (GRAVY index) were measured for each FBN after removal of the chloroplast transit peptide using the ProtParam tool (ExpPASy).

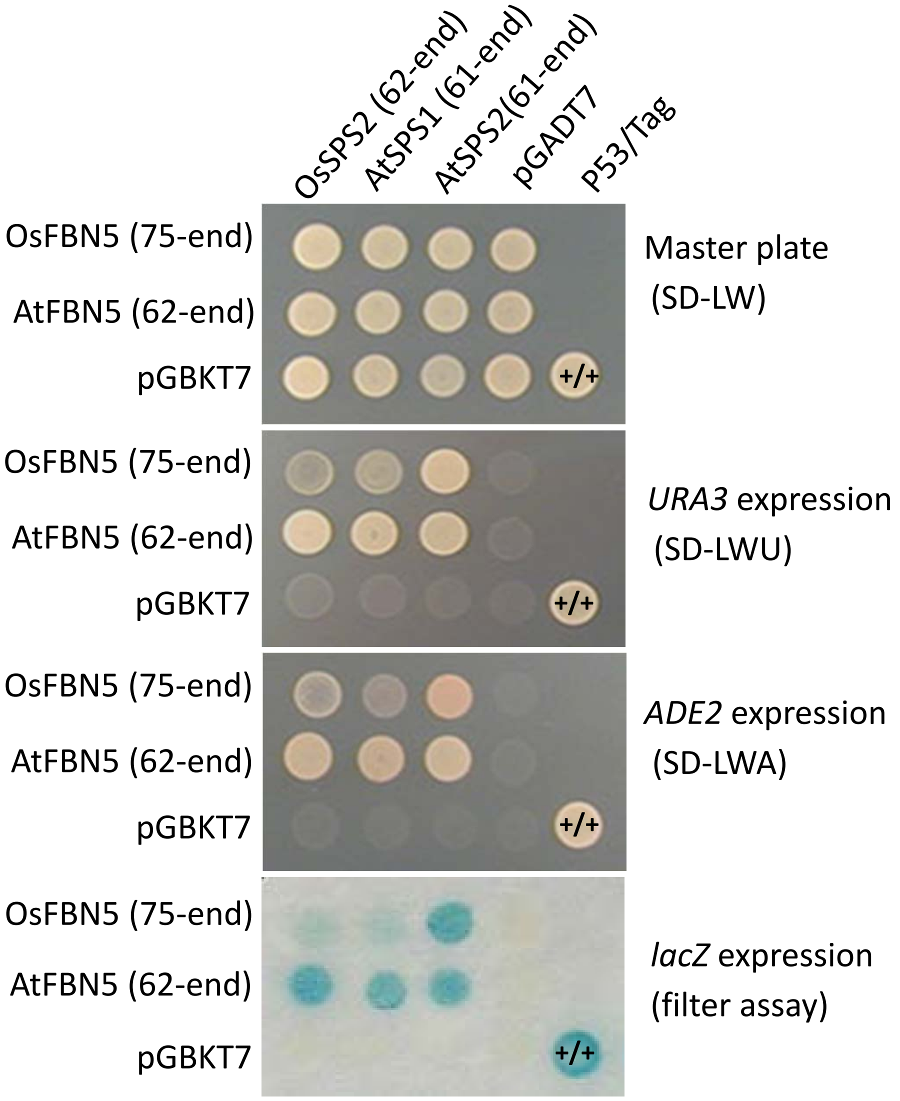

Supplementary Figure 3. Yeast two-hybrid assay for interaction of OsFBN5 and AtFBN5 with SPS enzymes from rice and Arabidopsis. OsFBN5 and AtFBN5 mature polypeptides were fused to the GAL4 DNA-binding domain. Mature forms of OsSPS2, AtSPS1 and AtSPS2 were fused to GAL4 DNA-activation domain. Transformed yeasts (PBN204) were dropped onto selective medium lacking Leu and Trp (SD-LW) or selective medium also lacking Ura (SD-LWU) or Ade (SD-LWA). β-Galactosidase activity in each colony was tested on SD-LWA medium. Positive control, yeast transformed with the P53 bait plasmid and Tag prey plasmid. Negative control, yeast transformed with the parental bait vector (pGBKT7) and prey vector (pGADT7). Blue and white colonies indicate interaction and no interaction, respectively.

```
AtSPS1   1   MMTSCRNIDLGTMMMACGCG------RRQFPSLAKTVCKFTSSNRS-----YGGLVGSCKAVPTKSKEISLLNGIGQ--S
ATSPS2   1   MMMSCRNIDLGTSVLDHSCSSSSTSRRFLFGNSSKTVCMIGGRSCVGNLVFLRRDLATCRAVPAKSKENSLVNGIGQDQT
OsSPS2   1   -------------MLSVSCPRVYMSRKALDFGQLASCRCRWAGRSGMRVAPRRRMPCVCFVASPSQPGLAAVDVPAEAIS
OsSPS3   1   ---MAAPSSLASSSHLSRRATAAASPSIPPPSPPPPPQRLRCGWVGRAAPPTRRAPGVCSVVSPSKPGVAAVDVPAATIP

AtSPS1   68  QTVSFDLKQESKQPISLVTLFELVAVDLQTLNDNLLSIVGAENPVLISAAEQIFGAGGKRMRPGLVFLVSHATAELAGLK
ATSPS2   81  VMETLNLRQESRKPISLETLFEVVADDLQRLNDNLLSIVGAENPVLISAAEQIFSAGGKRMRPGLVFLVSRATAELAGLK
OsSPS2   68  SARTT-TMIPER--ISVSSLLEVVSDDLLKLNNNLKSLVGAENPVLVSAAEQIFGAGGKRLRPALVFLVSRATAELAGLL
OsSPS3   78  DAAATGVGVAER--ISVSSLLEVVADDLLKLNNNLKSLVGAENPVLVSAAEQIFGAGGKRLRPALVFLVSRATAELAGLL

AtSPS1   148 ELTTEHRRLAEIIEMIHTASLIHDDVLDESDMRRGKETVHELFGTRVAVLAGDFMFAQASWYLANLENLEVIKLISQVIK
ATSPS2   161 ELTVEHRRLGEIIEMIHTASLIHDDVLDESDMRRGRETVHELFGTRVAVLAGDFMFAQASWYLANLENLEVIKLISQVIK
OsSPS2   145 ELTTEHQRLAEIIEMIHTASLIHDDVIDDSGMRRGKETIHQLYGTRVAVLAGDFMFAQSSWFLANLENIEVIKLISQVIK
OsSPS3   156 ELTTEHQRLAEIIEMIHTASLIHDDVIDDSGMRRGKETIHQLYGTRVAVLAGDFMFAQSSWFLANLENIEVIKLISQVIK

AtSPS1   228 DFASGEIKQASSLFDCDTKLDEYLLKSFYKTASLVAASTKGAAIFSRVEPDVTEQMYEFGKNLGLSFQIVDDILDFTQST
ATSPS2   241 DFASGEIKQASSLFDCDVKLDDYMLKSYYKTASLVAASTKGAAIFSKVESKVAEQMYQFGKNLGLSFQVVDDILDFTQST
OsSPS2   225 DFASGEIKQASTLFDCDVTLDDYLLKSYYKTASLLASSTRSAAIFSGVSTTICEQMYEYGRNLGLSFQVVDDILDFTQSA
OsSPS3   236 DFASGEIKQASTLFDCDITLDDYLLKSYYKTASLIAASTRSAAIFSGVSTAICEQMYEYGRNLGLSFQVVDDILDFTQSA

AtSPS1   308 EQLGKPAGSDLAKGNLTAPVIFALEREPRLREIIESEFCEAGSLEEAIEAVTKGGGIKRAQELAREKADDAIKNLQCLPR
ATSPS2   321 EQLGKPAANDLAKGNITAPVIFALENEPRLREIIESEFCEPGSLEEAIEIVRNRGGIKKAQELAKEKAELALKNLNCLPR
OsSPS2   305 EQLGKPAGSDLAKGNLTAPVIFALQDEPKLREIIDSEFSESDSLATAIDLVHRSGG------------------------
OsSPS3   316 EQLGKPAGSDLAKGNLTAPVIFALQDEPQLREIIDSEFSETNSLATAIELVHRSGGIKRAHELAREKGEIAIQSLQCLPR

AtSPS1   388 SGFRSALEDMVLYNLERID
ATSPS2   401 SGFRSALEDMVMFNLERID
OsSPS2   360 -------------------
OsSPS3   396 SEFRSTLENMVKYNLERID
```

Supplementary Figure 4. Sequence alignment of AtSPS1 (At1g78510, 406 aa), AtSPS2 (At1g17050, 419 aa), OsSPS2 (Os05G50550, 404 aa), and OsSPS3 (Os12g17320, 414 aa). The plastid targeting peptides of AtSPS1, AtSPS2, OsSPS2, and OsSPS3 (green box) were predicted by ChloroP. Amino acid sequences were aligned using ClustalW with default parameters. Identical residues are shaded in black; similar residues are shaded in gray. Sequence identity was calculated after removing the chloroplast targeting sequences using SIM (hppt://web.expasy.org/sim). AtSPS1 and AtSPS2 share 88% identical residues, while OsSPS2 and OsSPS3 share 92% identical residues. OsSPS2 showed 77.5% and 76.6% identity with AtSPS1 and AtSPS2, respectively. OsSPS3 showed 79.4% and 77.2% identity with AtSPS1 and AtSPS2, respectively.

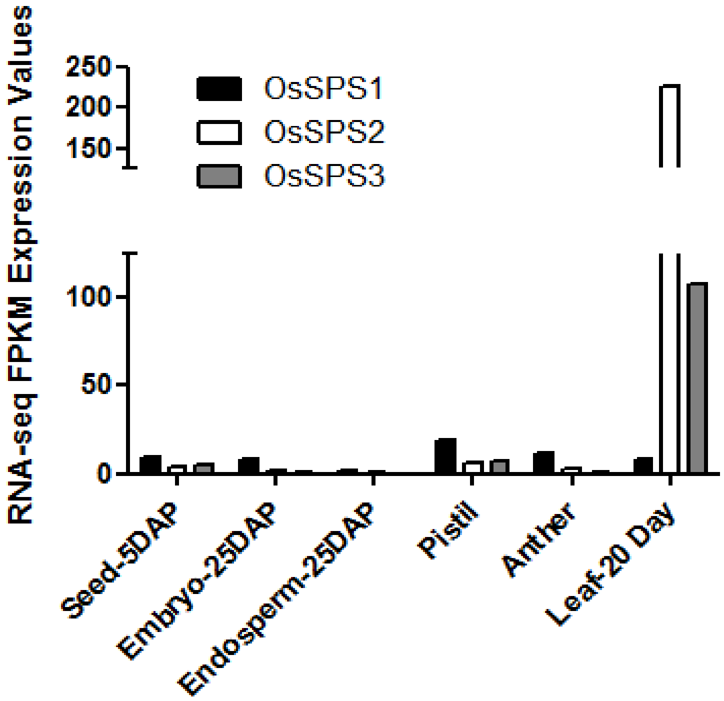

Supplementary Figure 5. Expression levels of *OsSPS1*, *OsSPS2,* and *OsSPS3* in different rice organs. Data were obtained from the Rice Genome Annotation Project. *OsSPS1* and *OsSPS2* are responsible for modification of the UQ-9 and PQ-9 solanesyl diphosphate moiety, respectively (Ohara et al., 2010). *OsSPS3* is presumed to be a paralog of *OsSPS2*.
