# Supplementary material for: Conserved Function of Fibrillin5 in the Plastoquinone-9 Biosynthetic Pathway in Arabidopsis and Rice
Source: Front Plant Sci. 2017 Jul 13;8:1197. doi: 10.3389/fpls.2017.01197 (PMC5507956; doi:10.3389/fpls.2017.01197)
Supplement: Supplementary file 2 [file Presentation_2.PDF]

Supplementary Table 1. Oligonucleotides used in this study

Gene	Use of primers	Primer
**Y2H**		
OsFBN5	F, Gateway cloning	CACCATGGCGGCGGTGCCTCCCGAG
OsFBN5	R, Gateway cloning	TCAAACTTCTGAGGGGTCTGCCCT
OsSPS2	F, Gateway cloning	CACCATGGCAGTTGATGTGCCAGCA
OsSPS2	R, Gateway cloning	TCAGTCAATCCTCTGAAGATTATA
**RT-PCR, Arabidopsis**		
AtFBN5	F	ATGACGAGTAACCTTTTCCAG
AtFBN5	R	TTAAGGTTTCTCTATTCTTTCC
OsFBN5	F	ATGGCGGCAGCTTCCGTCC
OsFBN5	R	TCAAACTTCTGAGGGGTCTG
Actin2	F	GGTGTCATGGTTGGGATGAA
Actin2	R	GATTCCTGGACCTGCCTCAT
**Genotyping, Arabidopsis**		
SALK_064597	AtLP	GAGACGAAATCTCGAAGACCC
SALK_064597	AtRP	AGAGGCATCGTATGGTGAATG
T-DNA	LBb1.3	ATTTTGCCGATTTCGGAAC
**Genotyping, Rice**		
ND8652,NG2517	LP	TTCTTGGACAATACCAAAATGATCAAAAC
ND8652,NG2517	RP	AGATTCATCAACGTATCTGACCAAAATGT
TOS17	LTR1	CCAGTCCATTGGATCTTGTATCTTGTATATAC
**RT-PCR, Rice**		
OsFBN5	F	GAACTATGATATGCTCCTTGCTAT
OsFBN5	R	GTAGTGTAACTGAACTTGCATGAG
OsSPS2	F	GCAGTTGATGTGCCAGCAGAG
OsSPS2	R	TCAGTCAATCCTCTGAAGATTATA
Ubiquitin5	F	GACTACAACATCCAGAAGGAGTC
Ubiquitin5	R	TCATCTAATAACCAGTTCGATTTC
**BiFC Assay**		
OsFBN5	F, Gateway cloning	CACCATGGCGGCAGCTTCCGTCCTCCTC
OsFBN5	R, Gateway cloning	AACTTCTGAGGGGTCTGCCCTCTC
OsSPS2	F, Gateway cloning	CACCATGTTGTCTGTGAGCTGCC
OsSPS2	R, Gateway cloning	GTCAATCCTCTGAAGATTATATTT
**Complementation**		
OsFBN5	F, Gateway cloning	CACCATGGCGGCAGCTTCCGTCCTCCTC
OsFBN5	R, Gateway cloning	TCAAACTTCTGAGGGGTCTGCCCT

Supplementary Table 2. Segregation analysis in the progeny of self-pollinated *Osfbn5-1* and *Osfbn5-2* heterozygous plants. Genotypes of progeny plants were determined by genomic DNA PCR using *OsFBN5*- and Tos17-specific primers.

**Parent line**	**Observed/expected genotype of progeny in % (Observed/analyzed plants in number)**		
OsFBN5/Osfbn5-1	*OsFBN5/OsFBN5*<br>26.9/25 (101/375)	*OsFBN5/Osfbn5-1*<br>48/50 (180/375)	*Osfbn5-1/Osfbn5-1*<br>25.1/25 (94/375)
OsFBN5/Osfbn5-2	*OsFBN5/OsFBN5*<br>26.2/25 (93/355)	*OsFBN5/Osfbn5-2*<br>48.2/50 (171/355)	*Osfbn5-2/Osfbn5-2*<br>25.6/25 (91/355)
